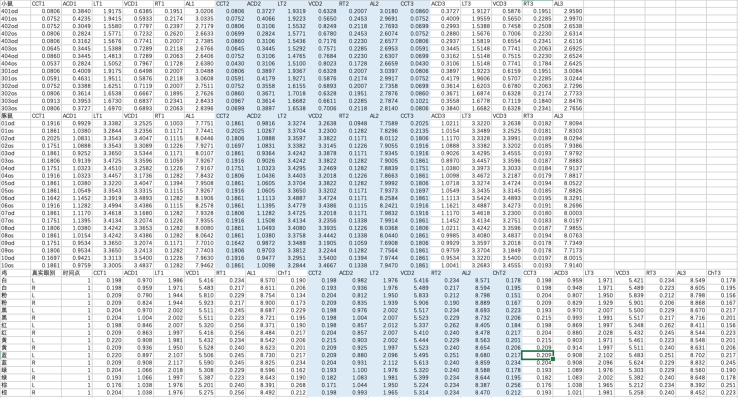# Biometric measurement with a commercially available swept-source optical coherence tomography in myopia model species

**DOI:** 10.1016/j.heliyon.2022.e12402

**Published:** 2022-12-17

**Authors:** Tian Han, Yuliang Wang, Yangyi Huang, Xun Chen, Xingxue Zhu, Yang Shen, Xingtao Zhou

**Affiliations:** a Department of Ophthalmology, Eye and ENT Hospital of Fudan University, Shanghai, China; b NHC Key Laboratory of Myopia (Fudan University), Shanghai, China; c Research Center of Ophthalmology and Optometry Shanghai, Shanghai, China; d Shanghai Engineering Research Center of Laser and Autostereoscopic 3D for Vision Care (20DZ2255000), Shanghai, China

**Keywords:** Optical coherence tomography (OCT), Biometric measurement, Axial length (AL), Myopia

## Abstract

**Background:**

Biometric parameters play an important role in studies on myopia. This study aimed to explore the application of a commercially available two-dimensional visualized swept-source optical coherence tomography (OCT) system, for *in vivo* biometric measurement in multiple myopia model species.

**Methods:**

In this study, chickens, guinea pigs, and C57BL/6 mice underwent eye imaging with the commercially available OCT (CASIA2), and the original images were used to calculate the central corneal thickness, anterior chamber depth (ACD), lens thickness (LT), vitreous chamber depth (VCD), and axial length (AL). The retinal thickness and choroidal thickness were also calculated in chicken eyes. The repeatability of the biometric measurement outcomes was analyzed.

**Results:**

Excellent repeatable AL measurements were obtained for all three species, with an intraclass correlation coefficient (ICC) of ≥0.941 and a within-subject standard deviation of ≤0.055. Excellent repeatability was found in chicken eyes for ACD, LT, and VCD, with an ICC of ≥0.932; in guinea pig eyes for ACD and VCD, with an ICC of ≥0.934; and in mouse eyes for LT, with an ICC of ≥0.941.

**Conclusions:**

It is effective to use commercially available OCT to measure biometric parameters in chickens, guinea pigs, and C57BL/6 mice. This methodology could potentially increase the accuracy and efficiency of future myopia animal experiments.

## 1 Introduction

According to the global incidence and prevalence statistics, approximately 50% of young adults in the USA and Europe are affected by myopia, and the proportion in East Asia is 80%–90% [1]. Moreover, it is predicted that myopia will have affected 4.9  billion individuals globally by 2050 [2]. High myopia with long axial length (AL) increases the risk of retinal detachment, cataracts, glaucoma, and even blindness [1]. The burden of these complications has significant socioeconomic consequences [3]. Therefore, the mechanisms underlying myopia are currently a research hotspot. Biometric measurement with high levels of repeatability and accuracy is necessary for impactful experimental animal research on myopia.

The contact A-scan is the most classic and commonly used AL measurement method [4, 5]. However, several factors (i.e., force, measured orientation, spatial resolution) [6] influence the repeatability and accuracy of the obtained measurements. Previous studies have also explored the potential role of the optical low coherence reflectometry-based Lenstar optical biometer [6, 7, 8, 9, 10]. However, this methodology is potentially problematic for measuring orientation considering that the presentation mode for the evaluated outcomes is a non-visualized one-dimensional wave.

Two-dimensional visualized optical coherence tomography (OCT) provides more comprehensive information than the methods above. Previous studies have demonstrated the application and utility of custom-made OCT in measuring AL [11, 12, 13]. To our knowledge, no study has used commercially available OCT for effective AL measurement before. Since such methodology should be easy to popularize, this study explored the application of commercially available swept-source OCT for *in vivo* biometric measurement. CASIA2 (Tomey Corporation, Nagoya, Japan) is a commonly used high-resolution swept-source anterior segment OCT, which uses a long wavelength (1,310 nm) to image the entire human anterior chamber. It has high repeatability for human anterior segment parameters [14]. Since the deep scanning depth of CASIA2 is 13 mm [15], which is larger than the AL of small myopia model species, i.e., chickens, guinea pigs, and C57BL/6 mice [11, 12, 13]. We considered it meaningful to examine whether such commercially available OCT can be used to achieve effective AL measurements in myopia model species.

This study focused on the practicality and repeatability of implementing CASIA2 scanning for conducting biometric measurements in commonly used myopic model species, i.e., chickens, guinea pigs, and C57BL/6 mice.

## 2 Methods

### 2.1 Animals

All experimental, animal handling, and humane care procedures were conducted according to the specifications of the Animal Research and Ethics Committee affiliated with the Eye and ENT Hospital of Fudan University and in accordance with the Association for Research in Vision and Ophthalmology Statement for the Use of Animals in Ophthalmic and Vision Research. Experimental protocols were approved by the Animal Research and Ethics Committee affiliated with the Eye and ENT Hospital of Fudan University.

Animals were provided by the Department of Animals affiliated with the EENT Hospital, the Shanghai Medical College of Fudan University, and the Shanghai Academy of Agriculture Sciences. All animals were maintained under a 12 h light/dark cycle. Eight white Leghorn chickens (aged 10 days), 10 guinea pigs (aged 4 weeks), and 7 C57BL/6 mice (aged 28 days) were used to test the repeatability of biometric measurements, which was the primary outcome of the study. Eight eyes of 40-day-old white Leghorn chickens were evaluated to explore the maximum range of the AL measurement. Since OCT shows the details in intraocular structures, it might be possible to simultaneously detect diseases. To test disease detection with OCT, images of 2 morbid guinea pig eyes (aged 4 weeks) with peripheral anterior synechia and nuclear cataract were also obtained.

### 2.2 OCT measurement

In guinea pigs, measurements were obtained without anesthesia, while mice and chickens were anesthetized with tiletamine hydrochloride and zolazepam hydrochloride prior to measurement (VIRBAC, Carros, France). An eyelid opener was used to open the chicken eyelids to complete the procedure after administering oxybuprocaine hydrochloride eye drops (Santen, Osaka, Japan).

CASIA2 was used to acquire images. During scanning, the optical axis of the incident ray was required to pass through the vertex of the cornea vertically. The real-time images were displayed on both vertical and horizontal image-monitoring screens. A researcher held and adjusted the animals to ensure that the irises were horizontal to the reference line in both the vertical and horizontal image-monitoring screens. CASIA2 scanning lasts approximately 2.4 s (50,000 A-scans per second). Note that it was necessary to maintain the hands stable during scanning until the machine beeped. Another researcher operating the machine was responsible for ensuring that the center of the incident ray was located at the vertex of the cornea and that the retina image was oriented in the right direction. Each eye was measured thrice.

The biometric measurements were completed manually by one investigator (YH) after obtaining one image from each measurement. The original OCT images were used to calculate the corrected geometrical lengths from the optical path lengths. Pixel density in depth direction differs across machines. For our CASIA2, the pixel density is 0.007531 mm/pix. An equation (geometrical length = pixel length ∗ pixel density) was used to calculate the lengths in millimeter. The obtained measurement data included central corneal thickness (CCT), anterior chamber depth (ACD), lens thickness (LT), vitreous chamber depth (VCD), and AL. Retina thickness (RT) and choroid thickness (ChT) could also be calculated only in chicken models since the boundary was not sufficiently clear for conducting effective measurements in guinea pigs and C57BL/6 mice. Owing to the blurry outlines between the retinal pigment epithelium (RPE) and choroid, ChT was measured starting from the upper outline of the RPE. For animals with long ALs beyond the measurement range, two images were captured: one for the posterior segment (with the lens, vitreous body, and retina), and one for the anterior segment. In the present study, we defined AL as the distance between the anterior surface of the cornea and the retina [7, 10, 16]. The refractive indices for the calculation of dimensional variables based on prior studies are as follows: for mice [13, 17, 18, 19], 1.4015 for the cornea, 1.3336 for the aqueous, y = 0.0005 age in day +1.557 for the lens, and 1.3329 for the vitreous; for guinea pigs [12, 20], 1.376 for the cornea, 1.335 for the anterior chamber, 1.401 for the lens, and 1.336 for the vitreous chamber; for chickens [11, 21], 1.369 for the cornea, 1.335 for the anterior chamber, 1.437 for the lens, 1.335 for the vitreous chamber, and 1.351 for the retina and choroid.

### 2.3 Statistical analysis

R statistical software (version 4.0.2; The R Project for Statistical Computing, Vienna, Austria) was used to conduct all statistical analyses. The normal distribution of the data was verified using the Shapiro–Wilk test. All parameters satisfied the normal distribution and are expressed as means ± standard deviations. To evaluate the repeatability of 3 measurement results, we calculated within-subject standard deviation (Sw), test–retest repeatability (TRT), within-subject coefficients of variation (CoV), and intraclass correlation coefficients (ICC). TRT was equal to 2.77 × Sw, demarcating the expected upper limit of 95% of the difference in the measured values. CoV was calculated as the Sw/arithmetic mean × 100%. A lower CoV indicated higher repeatability. Most researchers believe that devices with a CoV of <10% show high repeatability and that a CoV of <5% indicates an excellent degree of repeatability. ICC values greater than 0.9, between 0.75 and 0.9, between 0.5 and 0.75, and lower than 0.5 are considered to indicate excellent, high, moderate, and poor reliability, respectively. For the sample size calculation, we considered an ICC value of ≥0.9 based on our experience. Thus, a sample of 3 eyes would be sufficient to confirm non-inferiority with a power of ≥90% at a 15% significance level.

## 3 Results

**Figure 1:**
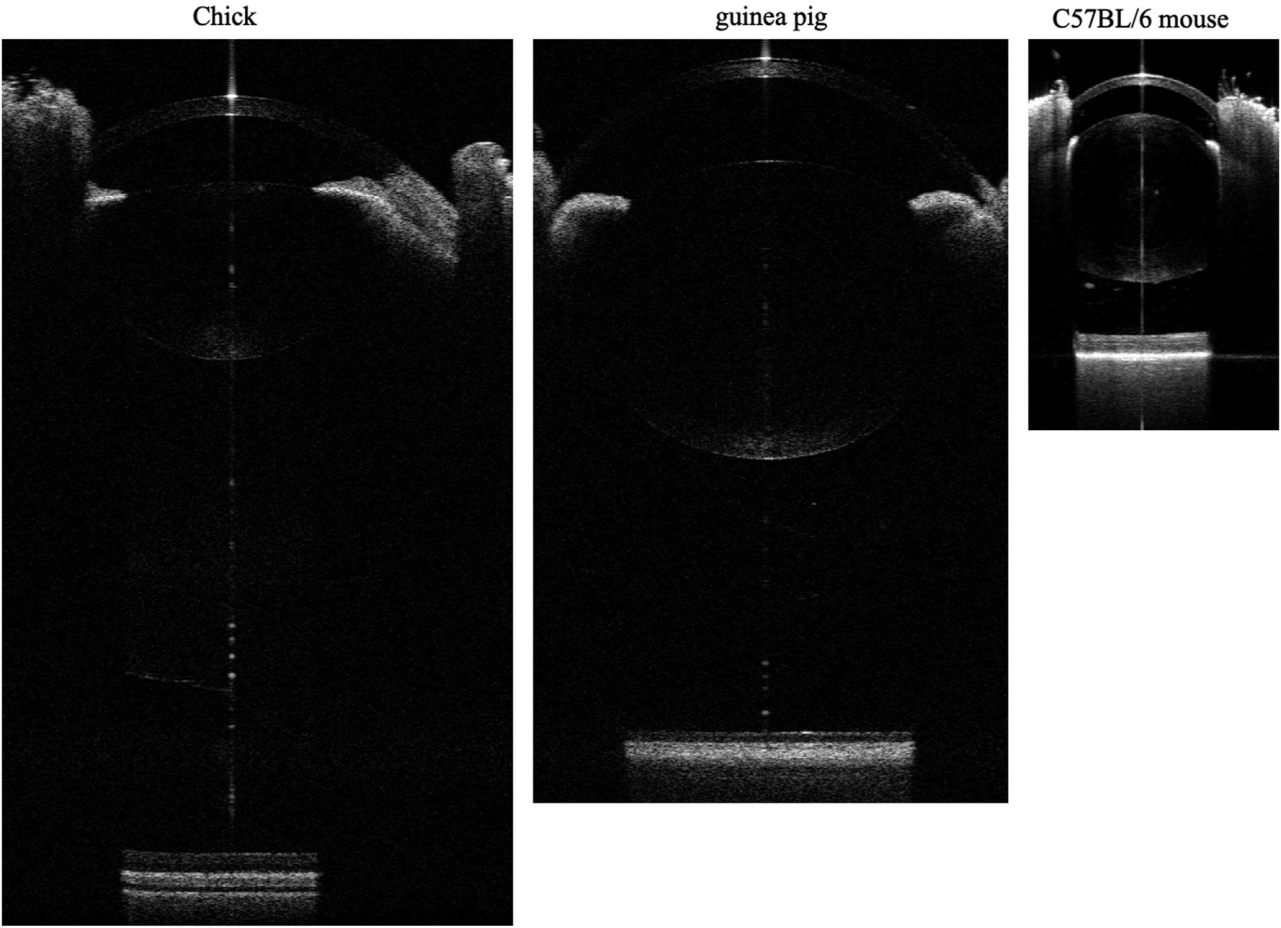
OCT images of the entire eyes of a chicken, guinea pig, and C57BL/6 mouse. The images are modified in the transverse direction.

OCT images of the entire eye, including the cornea, anterior chamber, lens, vitreous chamber, and retina/sclera, were successfully obtained (Figure 1). The measurement repeatability and measurement values are shown in Table 1 for chicken eyes, in Table 2 for guinea pig eyes, and in Table 3 for C57BL/6 mouse eyes.

**Table 1:** Distribution of the repeatability and values of the measurement in chicken eyes.

OD	Mean (mm)	SD (mm)	Sw (mm)	TRT (mm)	CoV (%)	ICC	95% CI (Lower limit)	95% CI (Upper limit)
CCT	0.200	0.013	0.006	0.016	2.332	0.903	0.747	0.975
ACD	0.941	0.090	0.015	0.042	1.296	0.987	0.962	0.997
LT	1.997	0.046	0.017	0.047	0.687	0.935	0.824	0.983
VCD	5.444	0.176	0.016	0.045	0.240	0.996	0.989	0.999
AL	8.582	0.135	0.025	0.069	0.234	0.984	0.955	0.996
RT	0.237	0.013	0.008	0.023	2.776	0.819	0.568	0.950
ChT	0.198	0.034	0.014	0.040	5.817	0.919	0.783	0.979
OS	Mean (mm)	SD (mm)	Sw (mm)	TRT (mm)	CoV (%)	ICC	95% CI (Lower limit)	95% CI (Upper limit)
CCT	0.202	0.009	0.007	0.018	2.583	0.729	0.411	0.922
ACD	0.947	0.079	0.018	0.049	1.496	0.977	0.934	0.994
LT	1.997	0.049	0.019	0.052	0.757	0.932	0.816	0.982
VCD	5.517	0.179	0.020	0.055	0.289	0.994	0.983	0.999
AL	8.663	0.141	0.026	0.072	0.241	0.984	0.955	0.996
RT	0.231	0.015	0.012	0.033	4.136	0.722	0.398	0.919
ChT	0.204	0.020	0.008	0.022	3.160	0.928	0.807	0.981

OD: oculus dextrus, Sw: within-subject standard deviation, TRT: test–retest repeatability, CoV: within-subject coefficients of variation, ICC: intraclass correlation coefficients, OS: oculus sinister, CCT: central corneal thickness, ACD: anterior chamber depth, LT: lens thickness, VCD: vitreous chamber depth, AL: axial lengths, RT: retina thickness, ChT: choroid thickness.

**Table 2:** Distribution of the repeatability and values of the measurement in guinea pig eyes.

OD	Mean (mm)	SD (mm)	Sw (mm)	TRT (mm)	CoV (%)	ICC	95% CI (Lower limit)	95% CI (Upper limit)
CCT	0.183	0.009	0.011	0.032	6.222	0.201	0.123	0.615
ACD	1.031	0.069	0.022	0.061	2.151	0.952	0.879	0.985
LT	3.388	0.062	0.043	0.119	1.265	0.771	0.519	0.922
VCD	3.360	0.120	0.045	0.125	1.339	0.934	0.839	0.979
AL	7.962	0.146	0.051	0.141	0.639	0.943	0.860	0.982
OS	Mean (mm)	SD (mm)	Sw (mm)	TRT (mm)	CoV (%)	ICC	95% CI (Lower limit)	95% CI (Upper limit)
CCT	0.186	0.009	0.011	0.030	5.887	0.193	0.130	0.608
ACD	1.037	0.073	0.018	0.051	1.782	0.970	0.924	0.991
LT	3.389	0.063	0.028	0.079	0.839	0.904	0.772	0.970
VCD	3.326	0.100	0.027	0.076	0.826	0.965	0.911	0.989
AL	7.938	0.140	0.038	0.106	0.484	0.965	0.911	0.989

OD: oculus dextrus, Sw: within-subject standard deviation, TRT: test–retest repeatability, CoV: within-subject coefficients of variation, ICC: intraclass correlation coefficients, OS: oculus sinister, CCT: central corneal thickness, ACD: anterior chamber depth, LT: lens thickness, VCD: vitreous chamber depth, AL: axial lengths.

**Table 3:** Distribution of the repeatability and values of the measurement in C57BL/6 mouse eyes.

OD	Mean (mm)	SD (mm)	Sw (mm)	TRT (mm)	CoV (%)	ICC	95% CI (Lower limit)	95% CI (Upper limit)
CCT	0.081	0.008	0.007	0.018	8.178	0.704	0.306	0.933
ACD	0.347	0.035	0.018	0.051	5.293	0.877	0.668	0.971
LT	1.678	0.168	0.018	0.050	1.083	0.995	0.983	0.999
VCD	0.697	0.062	0.046	0.128	6.641	0.744	0.407	0.936
AL	2.803	0.152	0.055	0.152	1.954	0.941	0.829	0.987
OS	Mean (mm)	SD (mm)	Sw (mm)	TRT (mm)	CoV (%)	ICC	95% CI (Lower limit)	95% CI (Upper limit)
CCT	0.069	0.013	0.008	0.022	11.217	0.828	0.563	0.959
ACD	0.360	0.053	0.018	0.051	5.099	0.945	0.839	0.988
LT	1.693	0.174	0.023	0.064	1.365	0.992	0.975	0.998
VCD	0.674	0.080	0.031	0.085	4.568	0.932	0.803	0.985
AL	2.797	0.154	0.037	0.104	1.341	0.973	0.918	0.994

OD: oculus dextrus, Sw: within-subject standard deviation, TRT: test–retest repeatability, CoV: within-subject coefficients of variation, ICC: intraclass correlation coefficients, OS: oculus sinister, CCT: central corneal thickness, ACD: anterior chamber depth, LT: lens thickness, VCD: vitreous chamber depth, AL: axial lengths.

In chicken eyes, the ACD, LT, VCD, and AL measurements revealed excellent repeatability within the CASIA2 methodology. The CoV values were ≤1.496%, and all ICC values were ≥0.932. The AL outcomes were 8.582 ± 0.135 mm (oculus dextrus; OD) and 8.663 ± 0.141 mm (oculus sinister; OS), respectively. The Sw values for AL were within 0.026 mm, and the TRT was ≤0.072 mm. ChT showed a high degree of repeatability, with a CoV of ≤5.817% and an ICC of ≥0.919. The repeatability of the CCT and RT measurements was moderate, with an ICC of ≥0.722 (Table 1).

Excellent repeatability was shown for the ACD, VCD, and AL measurements of guinea pig eyes. The CoV values were ≤2.151%, and all ICC values were ≥0.934. The AL outcomes were 7.962 ± 0.146 mm (OD) and 7.938 ± 0.1401 mm (OS). The Sw values for AL were within 0.051 mm, and the TRT was ≤0.141 mm. The LT showed a high degree of repeatability, with a CoV of ≤1.265% and an ICC of ≥0.771. The repeatability of the CCT was poor, with an ICC lower than 0.201 (Table 2).

LT and AL measurements demonstrated excellent repeatability in C57BL/6 mouse eyes. The CoV values were ≤1.954%, and both ICC values were ≥0.941. The Sw values for AL were within 0.055 mm, and the TRT was ≤0.152 mm. The ACD and VCD showed a high degree of repeatability, with a CoV of ≤5.293 mm and an ICC of ≥0.877. The repeatability of CCT was moderate, with an ICC of greater than 0.704 (Table 3).

**Figure 2:**
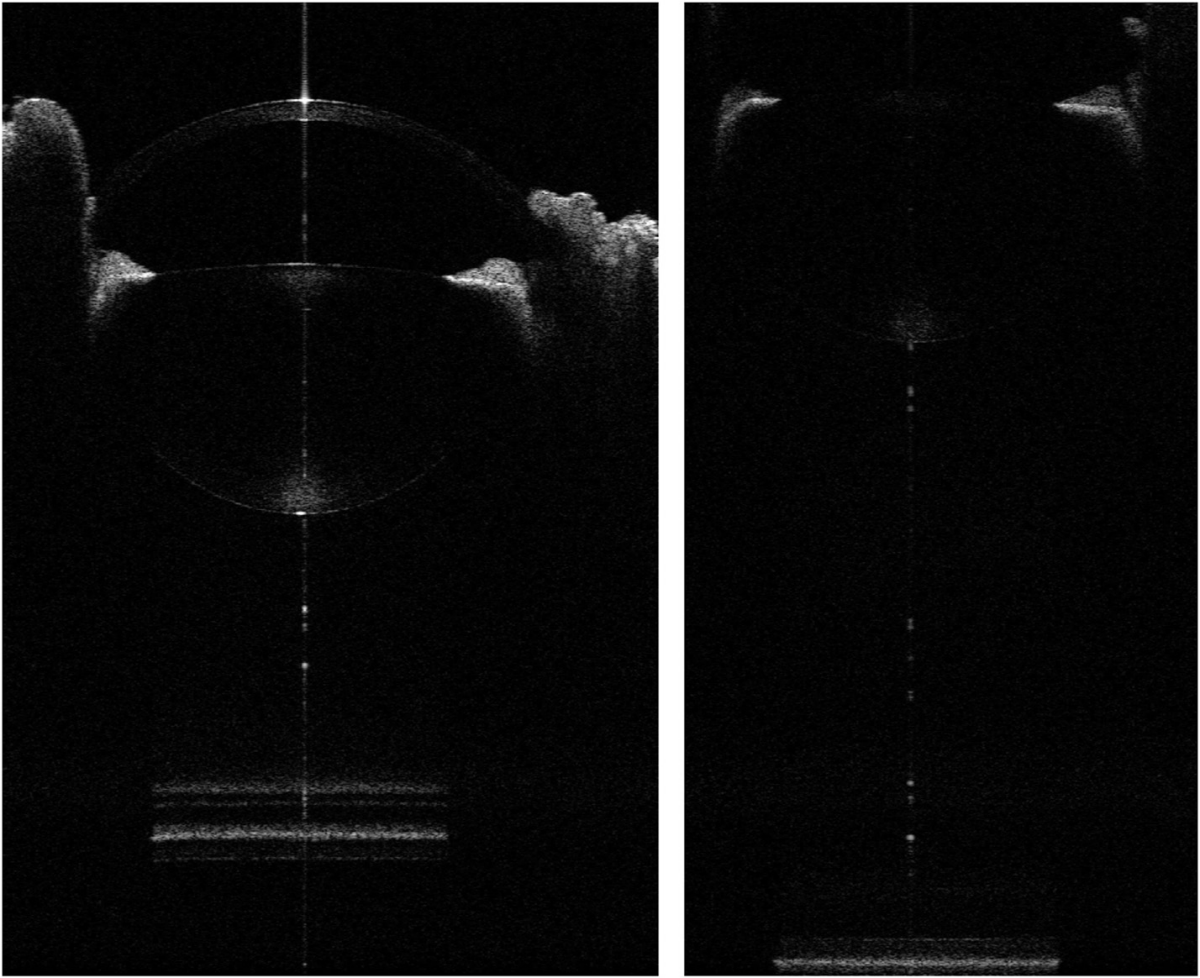
OCT images of the anterior segment (left panel) and posterior segment (right panel) from the same eye of a 40-day-old chick.

The average AL of the eight eyes of the 40-day-old chickens was 10.450 ± 0.217 mm. The maximum value of the eye with the longest AL among the eight eyes was 10.699 ± 0.026 mm Figure 2 depicts this measurement outcome.

**Figure 3:**
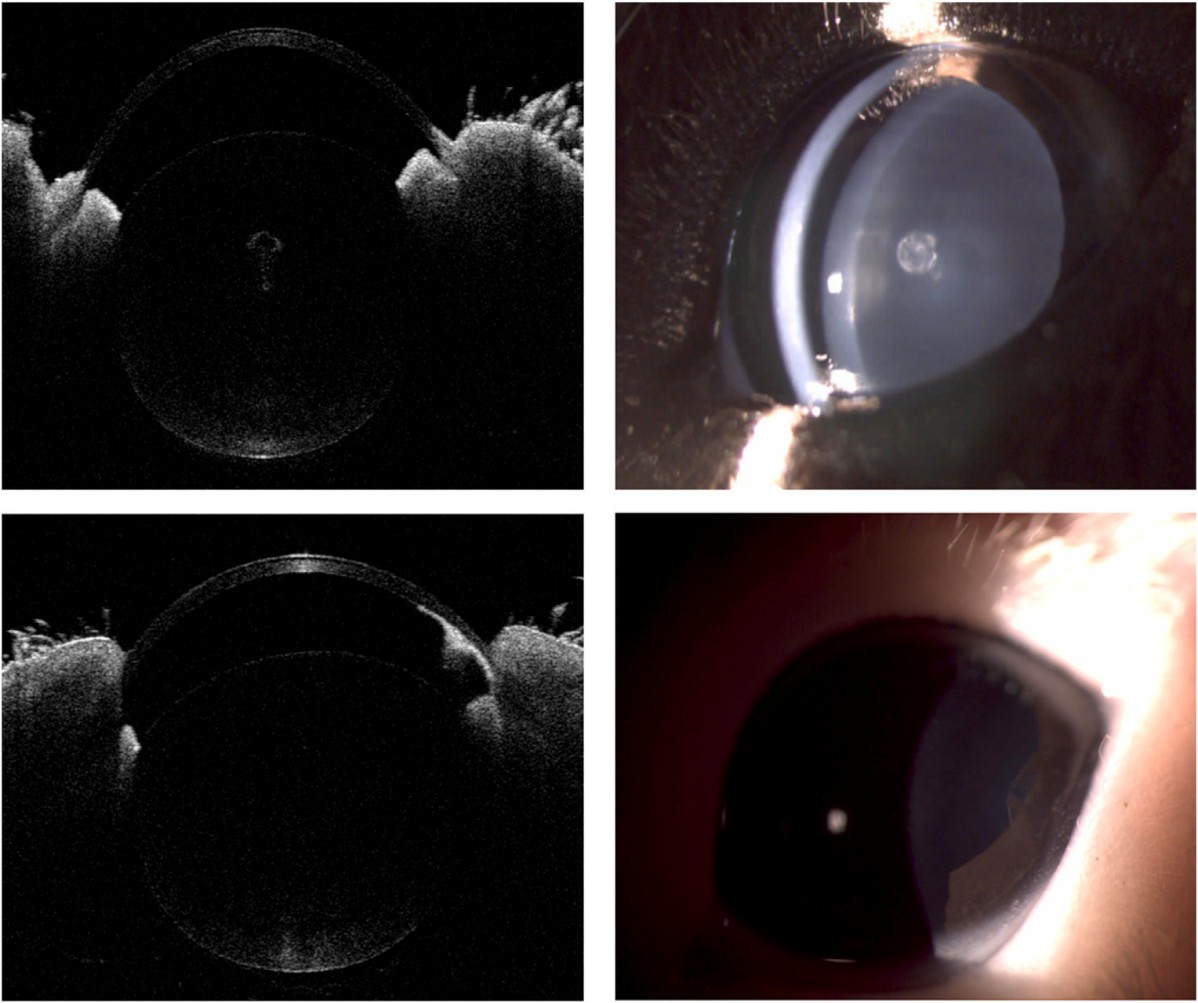
Images of two morbid guinea pig eyes. Opacity is detected in the nuclear zone of the lens under OCT and slit lamp examination (upper panel). Peripheral anterior synechia is found under OCT and slit lamp examination (lower panel).

Images of the two morbid guinea pig eyes with peripheral anterior synechia and nuclear cataracts are shown in Figure 3.

## 4 Discussion

OCT has been widely used in human disease diagnostics and medical treatments. The application of swept-source OCT enables the visualization of anatomical details of a longitudinal section through the anterior segment of human eyes or the entire eye in animals such as chickens, guinea pigs, and mice. In particular, the fast speed of imaging enables measuring the AL in these animals. To the best of our knowledge, this is the first study to evaluate the practicality and repeatability of a commercially available and mature OCT method for obtaining biometric measurements in different myopia model species.

OCT images of the entire eye, including the cornea, anterior chamber, lens, vitreous chamber, and retina/sclera, were successfully obtained in the current study. Excellent repeatable AL measurements were obtained in all three model species, and all ICC values were higher than 0.9, demonstrating the feasibility of CASIA2 to measure AL in these animals.

**Figure 4:**
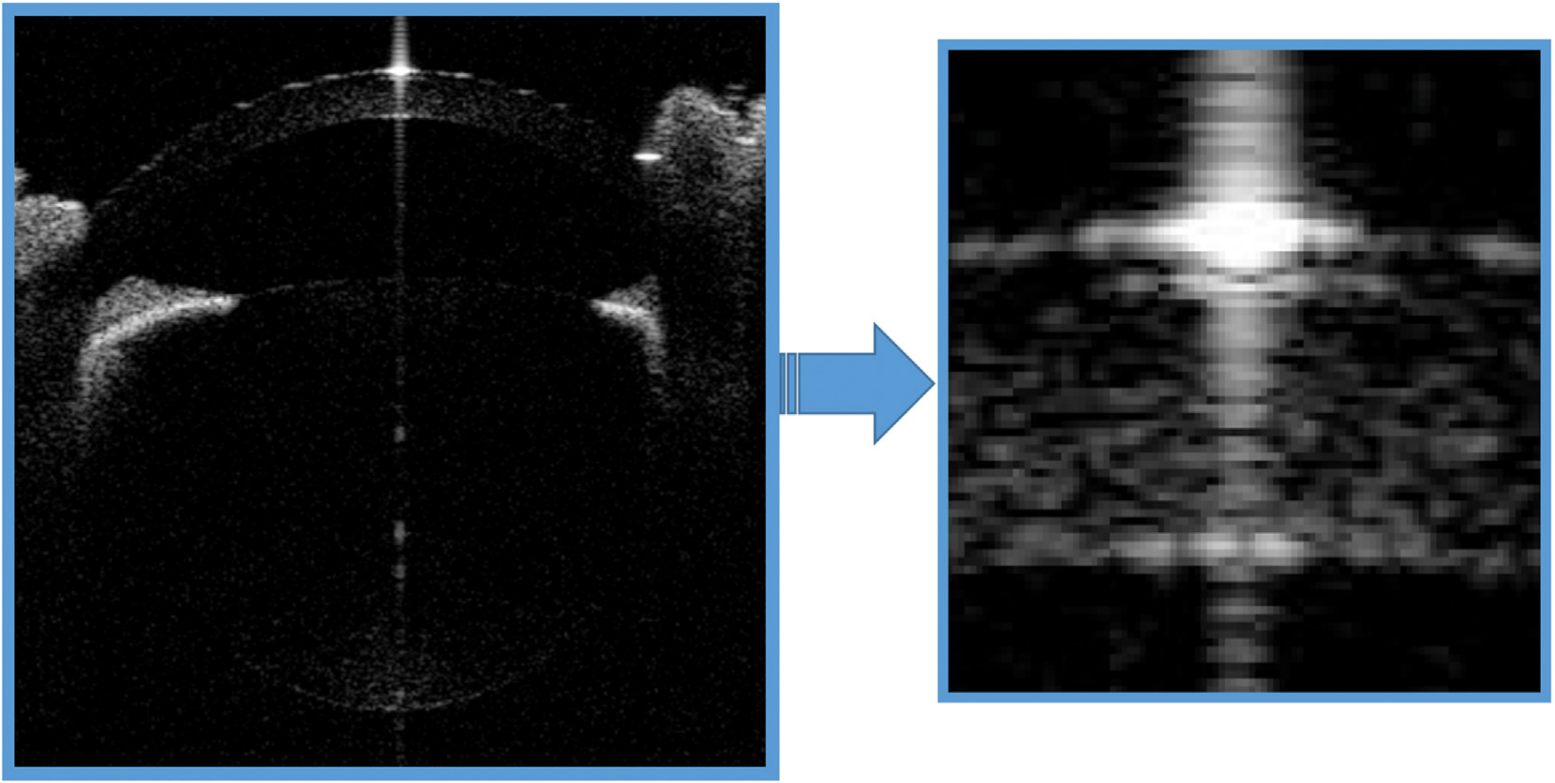
Blurred boundaries with low contrast in a chick's cornea. Although the boundary lines appear to be clear in the figure below (left panel), they appear blurred in the enlarged image (right panel).

All ocular dimensions showed excellent or high repeatability, except CCT in all three model species and RT in chicken eyes. Apparently, higher repeatability was found in tissues with longer lengths than in tissues with shorter lengths in all three model species. The reason might be the distinction of the boundary line with low contrast (Figure 4), which might cause an approximately 2-pix (0.015 mm) calculation error. Although similar calculation errors existed in all ocular dimensions, they were more influential in tissues with shorter lengths than in tissues with longer lengths since the proportion of calculation errors on the measurement values were more significant. Repeatability could be improved by using a learnable despeckling technique and automatic layer segmentation in the future.

Similarly, the repeatability of the AL results among the three model species shared the rule that the CoV for guinea pigs was better than that for mice but worse than that for chickens. In a recent study [11], Yan et al. used a custom-made OCT to measure the CoV values for CCT, ACD, LT, VCD, RT, and ChT in chicken eyes and obtained values of 1.7, 0.3, 0.4, 0.2, 1.9, and 3.0, respectively. These results were similar to our findings. In addition, it is readily observable on our figures that the measurement resolution with CASIA2 in the present study was better than that with the custom-made OCT. Owing to the poor cooperation of the animals, there is a notable difference in repeatability compared with human outcomes [14]. We believe that repeatability can be promoted in the future by auxiliary tools to ensure that the animals remain immobile.

This study utilized three commonly used myopia model species [22]. The longest AL among the eight chicken eyes was 10.699 ± 0.026 mm and could be measured with CASIA2 in this study. Considering the different refractive indexes of different species, we recommend that the eyes of other model species with AL values of approximately 10 mm or lower can likewise be measured using CASIA2.

This study also found that OCT can help myopia researchers quickly screen out some types of pathological eyes, including eyes with nuclear cataracts and peripheral anterior iris adhesions. The efficiency of myopia research can be improved considerably by simplifying the process of animal screening. This is important because abnormal eyes severely interfere with experiment results, and the proportion of eyes with an abnormality can be as high as 45% in guinea pigs [23].

To solve the problem of the tilted anatomy of animal eyes. It is important to simultaneously adjust the iris plane in both the vertical and horizontal directions since the measurement of AL is only accurate when the optical axis of the incident ray is along the visual axis of the eye. Thus, although these examinations can be completed by one person with the assistance of a support device, the cooperation of two colleagues is recommended according to researcher experience.

It should be noted that the complicated and non-uniform refractive indices of animal eyes might affect AL measurements. The lens of guinea pigs and chickens exhibit changes in gradient indices in terms of refraction profiles, such that different surface and nucleus refractive indices have been found to increase slightly [24]. Additionally, the refractive index of the choroid uses the same index of the retina in chickens [11]. However, since self-control and interocular biometric evaluations are common in myopia studies, these errors can be considered to fall within a specific range. With the standard procedure recommended above, these outcomes could meet basic research needs and can thus be used in myopia studies.

Another limitation is that the measurements were not used for comparisons with other devices. In future studies, we will compare the obtained OCT measurements with A-scan methodology and strive to refine the refractive index, apply learnable despeckling technologies, and develop automatic layer segmentation.

In conclusion, the methods demonstrated in this study enable non-contact, stable, and efficient *in vivo* measurement of AL in three model species. It is efficient to use CASIA2 or other commercially available swept-source OCT devices to measure biometric parameters in guinea pigs, chickens, and C57BL/6 mice. This methodology could potentially increase the accuracy and efficiency of research regarding the mechanisms of myopia progression.

## Declarations

### Author contribution statement

Tian Han: Conceived and designed the experiments; Performed the experiments; Analyzed and interpreted the data; Contributed reagents, materials, analysis tools or data; Wrote the paper.

Yuliang Wang; Yangyi Huang: Performed the experiments; Analyzed and interpreted the data; Contributed reagents, materials, analysis tools or data; Wrote the paper.

Xun Chen; Xingxue Zhu: Performed the experiments; Contributed reagents, materials, analysis tools or data; Wrote the paper.

Yang Shen; Xingtao Zhou: Conceived and designed the experiments; Contributed reagents, materials, analysis tools or data; Wrote the paper.

### Funding statement

Tian Han was supported by National Natural Science Foundation of China for Young Scholars [Grant No. 82000929], the Shanghai Sailing Program [Grant No. 20YF1405000].

Dr Xingtao Zhou was supported by National Natural Science Foundation of China [Grant No. 81770955], Project of Shanghai Science and Technology [Grant No. 20410710100], Clinical Research Plan of SHDC [SHDC2020CR1043B], Project of Shanghai Xuhui District Science and Technology [2020-015 & XHLHGG202104], Shanghai Engineering Research Center of Laser and Autostereoscopic 3D for Vision Care [20DZ2255000], Construction of a 3D digital intelligent prevention and control platform for the whole life cycle of highly myopic patients in the Yangtze River Delta [21002411600].

### Data availability statement

Data included in article/supp. material/referenced in article.

### Declaration of interest’s statement

The authors declare no competing interests.

### Additional information

Supplementary content related to this article has been published online at [URL].

## Figures and Tables

**original data:**